# From Diabetes Care to Heart Failure Management: A Potential Therapeutic Approach Combining SGLT2 Inhibitors and Plant Extracts

**DOI:** 10.3390/nu14183737

**Published:** 2022-09-10

**Authors:** Micaela Gliozzi, Roberta Macrì, Anna Rita Coppoletta, Vincenzo Musolino, Cristina Carresi, Miriam Scicchitano, Francesca Bosco, Lorenza Guarnieri, Antonio Cardamone, Stefano Ruga, Federica Scarano, Saverio Nucera, Rocco Mollace, Irene Bava, Rosamaria Caminiti, Maria Serra, Jessica Maiuolo, Ernesto Palma, Vincenzo Mollace

**Affiliations:** 1 Pharmacology Laboratory, Institute of Research for Food Safety and Health IRC-FSH, Department of Health Sciences, University Magna Graecia of Catanzaro, 88100 Catanzaro, Italy; 2 Pharmaceutical Biology Laboratory, Institute of Research for Food Safety and Health IRC-FSH, Department of Health Sciences, University Magna Graecia of Catanzaro, 88100 Catanzaro, Italy; 3 Veterinary Pharmacology Laboratory, Institute of Research for Food Safety and Health IRC-FSH, Department of Health Sciences, University Magna Graecia of Catanzaro, 88100 Catanzaro, Italy; 4 Renato Dulbecco Institute, Lamezia Terme, 88046 Catanzaro, Italy

**Keywords:** diabetes, insulin resistance, lipid accumulation, inflammation, reactive oxygen species (ROS), SGLT2 inhibitors, nutraceutical supplementation, cardiovascular risk

## Abstract

Diabetes is a complex chronic disease, and among the affected patients, cardiovascular disease (CVD)is the most common cause of death. Consequently, the evidence for the cardiovascular benefit of glycaemic control may reduce long-term CVD rates. Over the years, multiple pharmacological approaches aimed at controlling blood glucose levels were unable to significantly reduce diabetes-related cardiovascular events. In this view, a therapeutic strategy combining SGLT2 inhibitors and plant extracts might represent a promising solution. Indeed, countering the main cardiometabolic risk factor using plant extracts could potentiate the cardioprotective action of SGLT2 inhibitors. This review highlights the main molecular mechanisms underlying these beneficial effects that could contribute to the better management of diabetic patients.

## 1. Introduction

Diabetes is a complex disease, characterised by chronic hyperglycaemia, which includes different subtypes of heterogeneous metabolic disorders, such as type 1 diabetes mellitus (T1DM) and type 2 diabetes mellitus (T2DM), gestational diabetes and monogenic diabetes syndromes [1].

In some forms of T1DM, the pathogenesis of the disease depends on the destruction of autoimmune β-cell that causes an absolute insulin deficiency, whereas a progressive loss of β-cell insulin secretion, frequently on the background of insulin resistance, leads to T2DM [1,2].

Clinically, the progressive loss of β-cell mass or function due to several genetic and environmental factors manifests as hyperglycaemia. In all subtypes of diabetes, it represents a risk for the development of chronic complications, although their progression may differ, as in the presence of different comorbidities (i.e., hyperlipidaemia) [1,3].

Among populations suffering from diabetes, cardiovascular disease (CVD) represents one of the most common causes of death. On the other hand, in the early stages of diabetes subtypes, glycaemic control and countering cardiovascular risk factors can reduce the CVD mortality rate [4].

Over the years, multiple pharmacological approaches aimed at controlling blood glucose concentrations in diabetic patients have been used. Starting from the use of insulin, the research progress in improving the pharmacotherapy of diabetes, through the discovery of metformin, sulfonylureas, and thiazolidinediones, failed to reduce cardiovascular events despite the beneficial effects on blood glucose regulation [5]. Differently, novel classes of antihyperglycaemic drugs, such as sodium-glucose cotransporter-2 (SGLT2) inhibitors (SGLT2i) and glucagon-like peptide-1 receptor agonist (GLP-1 RA), appeared more effective in preventing CV complications [6]. Starting from this evidence, some SGLT2i have even been approved to treat heart failure (HF) independently from the presence of diabetes [6,7,8]. Indeed, beyond the mechanisms underlying the benefit of SGLT2i on the cardio–renal axis [9], a recent study has shown that SGLT2i improve cardiovascular outcomes, affecting a small group of circulating intracellular proteins (e.g., IGFBP1 and TSMB10, implicated in cardioprotection and nephroprotection, respectively) which promote autophagic flux, reduce oxidative stress and inflammation, and stimulate repair and renewal in the heart and kidneys [10].

The purpose of this review is to focus on the molecular mechanisms underlying the therapeutic properties of SGLT2i in countering the detrimental effects of hyperglycaemia on cardiac function. On this background, we will overview the possible use of natural polyphenols in the prevention and management of diabetic cardiomyopathy (DCM) beyond glycaemic control.

## 2. SGLT2 Inhibitors and Molecular Mechanisms of Cardioprotection

SGLT2i are strongly favoured in diabetic patients with diagnosed HF or at risk of HF [11,12,13]. The choice of this therapeutic approach—preserving heart function—suggests the existence of specific mechanisms underlying these favourable effects based on the modulation of metabolism [14], haemodynamic parameters, electrolyte levels and neurohormonal activation [15]. In addition, cardiac protection is also mediated by the restoration of calcium homeostasis, anti-inflammatory, and antifibrotic effects.

### 2.1. SGLT2 Inhibitors and Modulation of Metabolism in Heart Tissue

**Figure 1:**
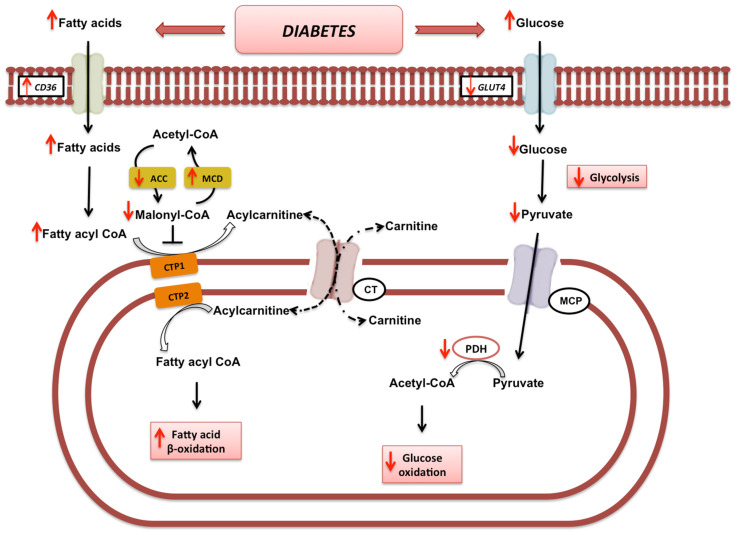
**Metabolic alterations in diabetic patients**. At the molecular level, insulin resistance causes an increase in both plasma glucose and serum insulin levels, which lead to a reduction in glucose transporter type 4 (GLUT4), and a decrease in glucose uptake. These events lead to a reduction in the glycolysis pathway and glucose oxidation caused by the downregulation of the pyruvate dehydrogenase complex (PDH). The hyperglycaemia is associated with the concomitant increase in hematic fatty acid concentration and the consequent upregulation of fatty acids uptake and oxidation due to the decreased levels of malonyl CoA and the consequent activation of carnitine palmitoyltransferase 1 (CPT1). CD36—cluster of differentiation 36; ACC—Acetyl-CoA carboxylase; MCD—Malonyl-CoA decarboxylase; CTP1—carnitine palmitoyltransferase 1; CTP2—carnitine palmitoyltransferase 2; MCP—mitochondrial pyruvate carrier; CT—carnitine translocase; PDH—pyruvate dehydrogenase complex; GLUT4—glucose transporter type 4.

As mentioned above, heart failure is associated with alterations in myocardial metabolic substrate flexibility [16]. In diabetic patients, the lack of glucose and fatty acid utilization, in turn, is associated with the gradual use of ketone bodies as a fuel source aimed to ensure cardiac function [17,18]. At the molecular level, this implies the downregulation of the fatty acid transporter carnitine palmitoyltransferase 1-*α* (CPT1-*α*), the downregulation of the glucose transporter type 4 (GLUT4), and the inhibition of the pyruvate dehydrogenase complex (PDH), mediated by the upregulation of pyruvate dehydrogenase kinase 4 (PDK4) [16], physiologically responsible for the entrance of carbohydrate intermediates into the Krebs cycle [19] (Figure 1).

In the insulin-resistant heart, impaired cardiac metabolism, characterised by deficient ATP production, can be prevented by empagliflozin [20], which promotes increased glucose oxidation associated with unchanged or lower ketone body oxidation rate. This suggests that SGLT2i-induced rise of circulating ketone body levels can represent an additional source of energy to support heart contractile function. In pigs, this beneficial metabolic effect afforded by empagliflozin was associated with an amelioration of left ventricle remodelling, and in humans, it was also confirmed in the presence of T2DM and coronary artery disease [15].

Thus, the restoration of the complex metabolic equilibrium in heart tissue, which is impaired under diabetes, represents an additional therapeutic goal of disease treatment.

### 2.2. SGLT2i-Mediated Anti-Inflammatory, Antifibrotic, and Antioxidant Effects on the Heart 

Circulating leukocytes are involved in the development of a chronic low-grade inflammatory state, characterized by increased circulating leukocyte-produced proinflammatory cytokines and decreased anti-inflammatory IL-10, which represents the main cause of enhanced cardiovascular risk in T2DM patients [21,22]. In diabetes and heart failure, pathogenic inflammatory cytokines, specifically IL-1β, are promoted by the activation of the Nod-like receptor (NLR) family pyrin domain-containing 3 (NLRP3) inflammasome. Although empagliflozin was shown to possess an analogue glucose-lowering power compared with the sulfonylurea, it can reduce IL-1β secretion mostly with an enhancement of serum β-hydroxybutyrate (BHB) and decreased serum insulin. Chronic inflammation and persistent oxidative stress lead to the development and progression of vascular proliferative diseases. The proinflammatory cytokine interleukin IL-17A induces oxidative stress and increases inflammatory signalling in human aortic smooth muscle cells through TRAF3IP2-mediated NLRP3/caspase-1-dependent mitogenic and migratory proinflammatory cytokines IL-1β and IL-18. It has been shown that the inhibition of SGLT2 in smooth muscle cells by empagliflozin decreased IL-17A/TRAF3IP2-dependent oxidative stress, NLRP3 expression, caspase-1 activation, and IL-1β and IL-18 secretion [23]. Additionally, empagliflozin can lessen serum uric acid levels, which is a potent activator of NLRP3 inflammasome. Thus, the inhibition of NLRP3 inflammasome, caused by the mentioned mechanisms, might contribute to explaining SGLT2i cardioprotective effects [24].

As proinflammatory cytokines promote the accumulation of neutrophils and macrophages into the lesion site, it has been demonstrated that, in DCM, this inflammatory environment also determines the release of growth factors, triggering fibroblast activation and the consequent development of fibrosis [25]. The prevention of remodelling and fibrotic processes after early and late treatment with empagliflozin was highlighted by the reduced increase in left ventricle mass and by the diminished cardiomyocyte cross-sectional area of myocardial infarction (MI) in diabetic hearts. Furthermore, SGLT2i significantly reduce the inflammatory response and infarct size in type 2 diabetic patients with acute myocardial infarction through a mechanism independent of glucose-metabolic control [26]. Moreover, in noninfarcted areas, empagliflozin-induced myocardial protection was confirmed by the reduced expression of specific markers of fibrosis, such as collagen 1 and procollagen [19].

It has been demonstrated that the sodium hydrogen exchanger-1 (NHE-1) may play a fundamental role in HF and diabetes since cardiac NHE-1 is upregulated in both conditions [27,28]. In rodent and rabbit models of diabetes [29], the inhibition of NHE-1 by SGLT2i and modulation of intramyocardial Ca^2+^ and Na^+^ fluxes seem to have a beneficial impact on diastolic myocardial function [29,30]. This effect can be connected to enhanced SERCA activity and to antifibrotic effects [31,32].

Although the NHE-1 inhibition affords cardioprotection in animal models, NHE-1 inhibitors failed in clinical practice, suggesting that the reduction in hyperactivation of the exchanger rather than the total inhibition of NHE-1 can represent the more appropriate therapeutic approach. In this view, the restoration of the basal activity might justify the cardioprotective effects of SGLT2i, similarly to other drugs modulating NHE1 activity through the control of its phosphorylation [33].

**Figure 2:**
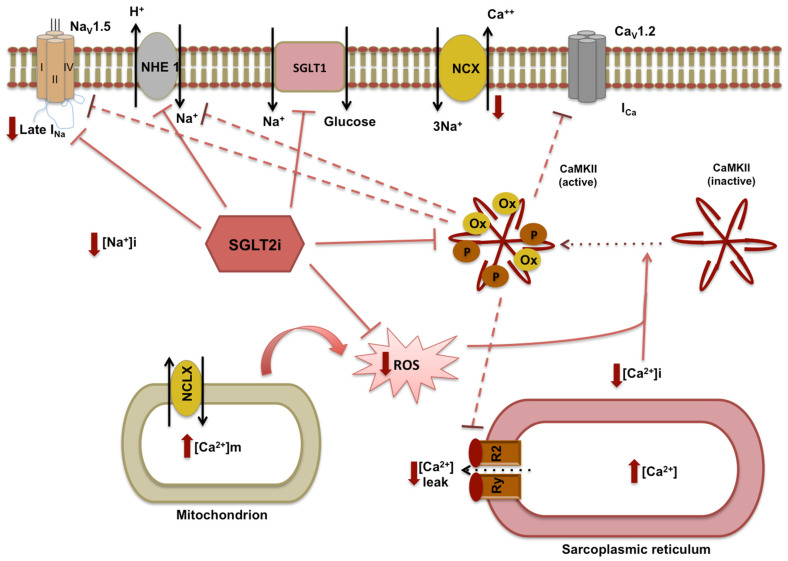
**Downregulation of NHE-1, modulation of intramyocardial Ca^2+^ and Na^+^ fluxes by SGLT-2 inhibitors.** The sodium hydrogen exchanger-1 (NHE-1) is upregulated in heart failure (HF) and diabetes. SGLT2i have a key role in the inhibition of NHE-1, sodium-glucose co-transporter 1 (SGLT1), and Na^+^/Ca^2+^ exchanger (NCX), preserving the calcium and sodium homeostasis and attenuating the oxidative Ca^2+^/Calmodulin-dependent kinase IIδ (CaMKII) activity. The modulation of Ca^2+^ and Na^+^ fluxes lead to a reduction in reactive oxygen species (ROS) and prevention of the cytosolic increase in calcium because of the mitochondrial and sarcoplasmic release caused by mitochondrial Na^+^/Ca^2+^ exchanger (NCLX) and ryanodine receptor 2 S(RyR2).

In turn, the reduced cytosolic Na^+^ and Ca^2+^_,_ caused by the modification of NHE-1 activity by empagliflozin, can cause an increase in mitochondrial Ca^2+^ [29,30], favouring the maintenance of cellular calcium handling and the prevention of the oxidative stress. Then, calcium homeostasis preservation provokes the attenuation of oxidative Ca^2+^/Calmodulin-dependent kinase IIδ (CaMKII) activity, downregulating NHE-1 and promoting the amelioration of diastolic and systolic functions [34] (Figure 2).

Further study indicated that oxidative stress and fibrosis development are suppressed by empagliflozin through the inhibition of TGF-beta, and the activation of Nrf2/ARE signalling [35].

## 3. SGLT2i and Cardiometabolic Risk Factors

It has been recognized that insulin resistance and atherogenic dyslipidaemia, characterised by the presence of low high-density lipoprotein (HDL)-cholesterol and high triglyceride levels [36,37], enhance the incidence of cardiovascular disease and diabetes mellitus. In this scenario, the positive impact of SGLT2i on cardiac tissue is due to counteracting the main cardiometabolic risk factors, which are also considered the cause of the metabolic activity dysregulation of other organs, such as liver and adipose tissue, which are directly involved in the control of lipid metabolism in the body.

Consequently, the modulation of specific molecular targets at those levels can also impact myocardial tissue function.

### 3.1. SGLT2i and Modulation of Lipid Metabolism

The most debated item related to the use of SGLT2i remains the effect on cholesterol low-density lipoprotein (cLDL) and triglycerides levels as, despite their proven cardioprotective effects, an increased level of cLDL emerged from several clinical studies. It has been hypothesised that the rise depends on the enhanced activity of lipoprotein lipase (LpL), independently of the turnover of circulating low-density lipoprotein (LDL), as confirmed by the lowered level of hepatic LDL receptors [38].

According to these discoveries, canagliflozin indirectly promotes LpL activity in the heart, white and brown adipose tissue, and skeletal muscle, also contributing to the decrease in triglycerides and very low levels of low-density lipoprotein (VLDL). On the other hand, the failed clearance of LDL is counterbalanced by a positive effect on LDL subclasses [39], further supporting the cardioprotective role of SGLT2i. In agreement with this evidence, canagliflozin administration contributed to very large high-density lipoprotein (VLHDL) and large high-density lipoprotein (LHDL) levels [40].

A meta-analysis of randomised clinical trials confirmed the amelioration of plasma lipid profile induced by SGLT2i in a dose-dependent manner [41]. In addition, although the most relevant effect concerns LDL subclass size and oxidation, canagliflozin [38] and dapagliflozin [42] have been shown to inhibit the hepatic synthesis of triglycerides through the downregulation of those enzymes responsible for the catabolism of fatty acids [43].

Among them, peroxisome proliferator-activated receptors (PPAR)-α and PPAR-γ appear to be interesting targets of SGLT2i because they might contribute to counteracting endothelial dysfunction too. Indeed, it has been demonstrated that their activation induces cholesterol removal from human macrophage foam cells [44]. Moreover, canagliflozin stimulates PPAR-α, promoting the uptake, utilization, and catabolism of fatty acids, whereas PPAR-γ activation ameliorates insulin sensitivity through the stimulation of fibroblast growth factor 21 (FGF21) and the cluster of differentiation 36 (CD36) enzyme [38].

In addition to the effects on plasma lipid profile and liver metabolism, SGLT2i therapy can exert its beneficial property by acting at the adipose tissue level. Indeed, although lower body weight in patients who have diabetes [45] has been correlated with the recovery of the caloric balance promoted by glucose excretion, it is also associated with the positive effects of SGLT2i on adipose tissue mass; indeed, its reduction is able to boost insulin sensitivity [46]. Moreover, SGLT2i-induced “visceral fat lowering” is associated with the reduction in inflammatory responses caused by dysfunctional adipocytes, which prevents lipotoxicity and energy imbalance.

Particularly, empagliflozin can ameliorate insulin sensitivity by counteracting inflammatory events promoted by M2 macrophage polarization through the browning of white adipose tissue, which promotes an increase in energy expenditure and thermogenesis [47,48]. Additionally, canagliflozin has been shown to improve insulin resistance by a reduction in visceral and ectopic fatty tissues [49], adipocytes number, and plasma lipids [47,48,50], whereas dapagliflozin improved adipocyte function by stimulating adiponectin production in obese patients with T2DM [51].

### 3.2. Haemodynamic and Vascular Effects

The origin of hypertension can be ascribed to the kidneys that are responsible for the control of haemodynamic parameters. As it occurs in diabetic patients, the injured kidneys determine an enhanced sympathetic activation and, consequently, vasoconstriction, sodium and water retention, and tachycardia, leading to a rise in blood pressure. The prolonged increase in sympathetic tone can ultimately lead to a decline in renal function and to an enhanced cardiac load contributing to the development of heart failure [52].

Thus, the favourable haemodynamic consequences of the SGLT2i action depend on the reduced preload and afterload at the ventricular level, and this final effect appears to be mediated by the regulation of several parameters, comprising osmotic diuresis, natriuresis, and plasma and interstitial fluid volume [53,54,55]. Overall, they contribute to relieving the renal load and reducing sympathetic nerve overactivation [50], lowering blood pressure [56,57]. Thus, the findings related to the action of SGLT2i on blood pressure highlight the dissociation of antihypertensive from antihyperglycemic effects [58]. Although some clinical studies suggest that SGLT2i do not directly affect haemodynamic parameters, it was highlighted that SGLT2i significantly reduce markers of tubular injury by certain mechanisms [59]. Indeed, at the molecular level, SGLT2i exert their beneficial effects also through the modulation of sodium hydrogen exchanger 3 (NH3), responsible for HCO_3_^-^ absorption at proximal tubule level in the absence of luminal glucose. It has been demonstrated that NH3 and SGLT2 can regulate each other; consequently, all those events characterizing diabetic conditions (i.e., low luminal glucose, the overactivation of the sympathetic nervous system, acidosis, oxidative stress, chronic kidney disease, and congestive heart failure (CHF)), contribute to inducing primarily NH3 and enhance SGLT2 expression [60]. Therefore, SGLT2 inhibition blocking sodium–hydrogen exchanger 3 (NHE3) may also prevent HCO_3_^-^ absorption and metabolic acidosis [56].

It has been recognized that luminal glucose and uric acid compete to bind Urate transporter 1 (URAT-1) for reabsorption at the proximal tubule level [61]. Consequently, SGLT2i, increasing luminal glucose concentration, causes the inhibition of URAT-1 [62], favouring renal uric acid clearance [63] and, at the same time, counteracting the detrimental effects of high concentrations of plasma urate, such as reactive oxygen species overproduction, inflammation, vascular proliferation, and renal damage [64]. In turn, the reduction in vascular and renal ROS contributes to restoring the physiological level [65] and function [66] of endothelial nitric oxide (NO), counteracting aortic stiffness [67,68,69]. Additionally, the normalization of NO production, probably caused by the inhibition of sodium–hydrogen exchanger 1 (NHE1), further contributes to reducing blood pressure [56]. Moreover, SGLT2i improve macro- and microvascular endothelial function [70] through the modulation of AT1R/NADPH oxidase/SGLT1 and two pathways, providing a promising strategy to maintain physiological endothelial function [69,71].

Recently, Mone et al. have identified a signature of microRNA functionally involved in endothelial function. In frail patients with heart failure with preserved ejection fraction and diabetes, the treatment with the SGLT2i caused a modification of these microRNA signatures, indicating a maintained endothelial function [72].

Finally, SGLT2i reduce frailty in diabetic and hypertensive patients by a reduction in mitochondrial generation of ROS species in endothelial cells [73].

## 4. DCM and Cardiometabolic Risk: Can Plant Extract Supplementation Support SGLT2i Action?

**Figure 3:**
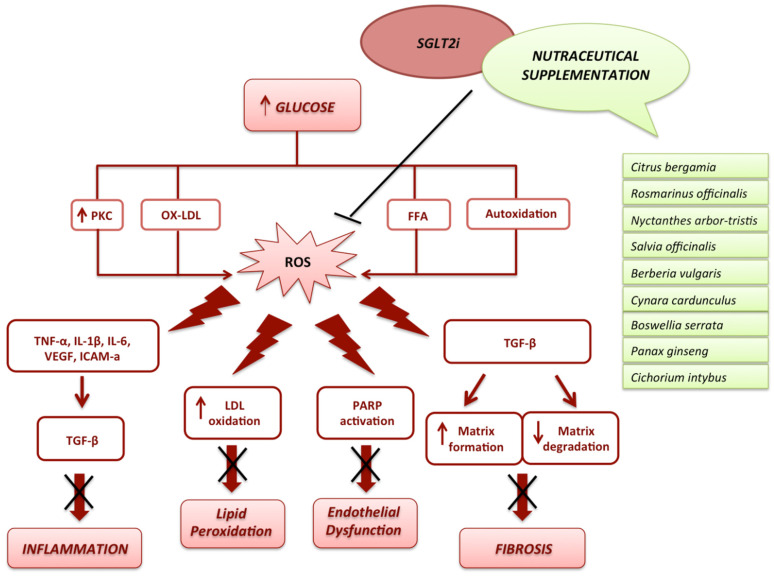
**Common cardioprotective effects of the plant extract supplementation and SGLT2 inhibitors.** The SGLT2i and several phytocomplexes show a similar action to counteract the ROS production caused by hyperglycemia. In particular, they play a key role in the inhibition of the inflammasome, leading to a reduction in inflammation and fibrosis. The downregulation of LDL oxidation and PARP activation lead to the reduction in lipid peroxidation and endothelial dysfunction. PKC—Protein kinase C; OX-LDL—Oxidized Low-Density Lipoprotein; FFA—Free fatty acids; ROS—reactive oxygen species; TNF-α—Tumour Necrosis Factor α; IL-1β—Interleukin 1 beta; IL-6—Interleukin 6; VEGF—Vascular-Endothelial Growth Factor; ICAM-a—intracellular adhesion molecule a, TGF-β—Transforming Growth Factor Beta; PARP—Poly (ADP-ribose) polymerase; LDL—Low-Density Lipoprotein.

Chemically, SGLT2 inhibitors are synthetic derivatives of phlorizin, the main phenolic glucoside in apple trees (roots, bark, shoots, and leaves) [74]. Despite its structural amelioration, aimed to selectively inhibit NHE-1 and promote renal glucose excretion, it has been proven that the phlorizin-rich extract can contribute to improving other aspects of diabetes as well as of other metabolic disorders, also ameliorating the most common cardiometabolic risk factors [75]. Recently, in accordance with this evidence, the peculiar value of several phytocomplexes in the prevention of CVD or as a supplement to most widespread therapies has emerged. This suggests a role for several plant extracts/nutraceuticals that, synergizing with SGLT2i or supporting their action through the modulation of glucose and lipid metabolism, might potentiate their cardioprotective effects under diabetes development (Figure 3).

### 4.1. Insulin Resistance

In vitro and in vivo studies demonstrated that several plant extracts can counteract insulin resistance through different mechanisms, such as the improvement of the glucose uptake process in tissues and the stimulation of pancreatic insulin secretion. 

In particular, glucose uptake regulation occurs principally through the enhancement of GLUT-4 expression and translocation into the cells, which can be mediated by several signalling pathways, including the AMP-activated protein kinase (AMPK), phosphoinositide 3-PI3K/Akt, PKC, and G protein–phospholipase C (PLC)–PKC pathways. 

*Cassia angustifolia Vahl* extract has been shown to improve GLUT-4 expression and turnover through the G protein–PLC–PKC signalling pathway and inositol 1,4,5-trisphosphate receptor (IP3R) [76].

*Morus alba* L. leaf extract counteracts insulin resistance and improves glucose metabolism through the activation of the IRS-1/PI3K/Glut4 signalling pathway at the muscular level. Additional mechanisms, due to anthocyanin extract of *Morus alba* L., are explicated by the activation of PI3K/Akt pathway [77] that regulates the basal expression of glucose-6-phosphatase (G6Pase) and phosphoenolpyruvate carboxyl kinase (PEPCK), two key rate-limiting enzymes of gluconeogenesis. Indeed, Akt activation inhibits gluconeogenesis through the phosphorylation of forkhead Box O1 (FOXO1) that renders the protein unable to translocate into the nucleus and to transcript G6Pase and PEPCK [78,79] proteins, thus blocking gluconeogenesis. In accordance with this evidence, FOXO1 downregulation has been shown to reverse hyperglycaemia in models of insulin resistance, and, in contrast, FOXO1 overactivation has been found to promote the insulin-resistant state [80,81,82].

*Cinnamomum cassia* extracts have also shown a significant improvement in glycaemic control in different animal models and patients with prediabetes dysfunction or diabetes [82]. The antidiabetic effect is due to different molecular mechanisms, including the control of insulin receptor (IR) phosphorylation, GLUT-4 expression and translocation, and the modulation of hepatic glucose metabolism, mediated by pyruvate kinase (PK) and phosphoenol pyruvate carboxykinase (PEPCK) activities [83].

Finally, *Rauwolfia serpentina* appears to counter insulin resistance, improving glucose metabolism, as proven by the reduction in total and glycosylated haemoglobin [76].

### 4.2. Antioxidant and Anti-Inflammatory Effects

Growing evidence shows that inflammation and oxidative stress can be counteracted by the active compounds of plant extracts.

*Boswellia serrata* extract [84], *Momordica charantia* L. fruit juice [85], and *Sclerocarya birrea* extract [86] have been shown to induce the overexpression of endogenous antioxidants, such as superoxide dismutase (SOD), catalase (CAT), and (glutathione) GSH, whereas *Nyctanthes arbor-tristis* L. leaf extract was able to suppress hyperglycaemia-induced oxidative stress and inflammation through the control of Nuclear factor kappa B (NF-kB) [87].

*Boswellia serrata* extract administration also reduced glucose, insulin, and cholesterol, in a T2DM rat model, preventing the hippocampal accumulation of Aβ 1-42 and glycogen synthase kinase-3β (GSK-3β) overexpression and counteracting excitotoxicity [84]. This beneficial response has been attributed to the downregulation of inflammatory mediators, such as TNF-α, IL-1β, and IL-6 and the amelioration of oxidative balance generated by the decrease in lipid peroxidation products and the upregulation of GSH and SOD levels [84].

*Cichorium intybus* L. extract improved insulin and glucose metabolism in high-fat diet-induced diabetic male C57BL/6 mice, and these effects were also mediated by the inhibition of NLRP3 inflammasome activation, leading to the decrease in IL-1β levels [88]. In adipose tissues, the inhibitory effect exerted on the NLRP3 inflammasome promoted the shift of M_1_ proinflammatory macrophages towards the M_2_ anti-inflammatory phenotype. In turn, this change was able to reduce the inducible nitric oxide synthase (iNOS) and TNF-α levels derived from M_1_ macrophages and to increase the expression of Arg-1 and IL-10, which are typical M_2_ markers [89].

*Panax ginseng* C.A. Meyer extract has been shown to ameliorate adipose inflammation in Ovariectomized female C57BL/6J mice through the reduction in the expression of proinflammatory cytokines CD68, TNF-α, and *Monocyte chemoattractant protein-1* (MCP-1) and the number of infiltrating inflammatory cells. In addition, it reduced MMP-2 and MMP-9 expression and activity, mRNA levels of *Vascular endothelial growth factor A* (VEGF-A), and Fibroblast growth factor 2 (FGF-2) [90].

*Salviaofficinalis* extract has a strong antioxidant activity. Indeed, experimental studies have shown that it induced the activation of glutathione peroxidase [91], preventing DNA damage in hepatocytes, and improved cellular superoxide scavenging power, enhancing catalase, glutathione peroxidase, glutathione-S-transferase, and superoxide dismutase in the pancreas [92].

In in vivo models of different inflammatory stimuli than diabetes, *Rosmarinus officinalis* has shown anti-inflammatory activity. This effect depended on the inhibition of NF-kB, leading to a reduced expression of cyclooxygenase-2 (COX-2) and iNOS, suggesting an antioxidant effect, too [93,94].

### 4.3. Lipid Metabolism

The modulation of lipid metabolism operated by plant extracts can be exerted at the liver, adipose tissue, and skeletal muscle level, with a consequent beneficial impact on those tissues and on the oxidative state of circulating lipids.

*Syzygium cumini* (L.) Skeels. and *Zingiber officinale* Roscoe extracts can positively modulate lipid metabolism upregulating PPARα and PPARγ [83,95]. Differently, the hypolipidemic effects of *Morus alba* L. leaf extract in diabetic rats have manifested with reduced levels of triglycerides, total cholesterol, and LDL with consequent inhibition of lipid accumulation in skeletal muscles [76]. In an alternative way, *Rauwolfia serpentina* improved lipid metabolism through the inhibition of 3-hydroxy-3-methylglutaryl-coenzyme A (HMG-CoA) reductase activity, suggesting an improvement in liver function [76].

### 4.4. Pleiotropic Effects of Plant Extracts in Counteracting Cardiometabolic Risk Factors and Heart Failure: Berberia Vulgaris and Citrus Bergamia

Several phytocomplexes in plant extracts have interesting therapeutic potential in the treatment of patients in whom the main cardiovascular risk factors tend to cluster, impairing the whole metabolism.

*Berberia vulgaris* and *Citrus Bergamia* extracts can represent peculiar examples of natural compounds that might be candidate supplements in an SGLT2i-based therapy.

*Berberia vulgaris*, historically used in the treatment of inflammations and high blood pressure [96,97], has been shown to have antihyperglycemic, hypolipidemic, and antioxidant effects; consequently, different parts of this plant, including fruits, leaves, and roots, have been used in traditional medicine for a long time [97,98].

Most of these effects have been attributable to berberine, which is able to reduce serum levels of triglycerides and total cholesterol, to increase the expression of cardiac fatty acid transport protein-1, fatty acid transport proteins, fatty acid beta-oxidase, and PPAR-γ [99]. Moreover, it ameliorates plasma lipoprotein profile through the increase in hepatic low-density lipoprotein receptor (LDLR) expression [100]. An additional mechanism responsible for lowering blood cholesterol levels includes the inhibition of absorption at the intestinal level with a reduction in enterocyte cholesterol uptake and secretion and the interference with intraluminal cholesterol micellization. Moreover, the upregulation of thermogenesis mediated by Uncoupling protein 1 (UCP1) and phosphorylated signal transducer and activator of transcription 3 (p-STAT3) in adipose tissue determines the reduction in proinflammatory cytokines (IL-6, TNF-α and MCP1) and infiltrating macrophages [101], thus contributing to improving insulin sensitivity [89]. Finally, studies carried out in dogs have shown that berberine improves cardiac output and reduces left ventricular end-diastolic pressure and systemic vascular resistance caused by an ischemic injury [102]. The amelioration of hemodynamic parameters and the hypotensive effect was also confirmed in humans [103], suggesting the role of this extract in combination therapy to counteract high blood pressure.

*Citrus bergamia* Risso et Poiteau (Bergamot) is an endemic plant, growing in Calabria (Southern Italy), that is characterized by a unique composition of flavonoids and glycosides in the extracts derived from the different parts of the fruit (i.e., essential oil, hydro-alcoholic extract, and fruit juice). An additional distinctive feature is the concentration of these bioactive compounds that, in the juice, is more abundant than in other citrus fruits [103,104].

Growing evidence suggests a protective role for bergamot extracts in the management of the metabolic syndrome. Although bergamot polyphenols have been shown to improve insulin resistance and glucose tolerance through the molecular mechanisms that still need to be clarified, several beneficial effects can be imputed to the pleiotropic anti-oxidative, anti-inflammatory, and lipid-lowering effects of bergamot extracts. This peculiar action has been studied in different experimental models (in vitro and in vivo) of metabolic dysfunctions that are commonly considered the main cardiometabolic risk factors.

The antioxidant effect of bergamot was first discovered by testing the activity of the nonvolatile fraction of the bergamot essential oil (BEO-NVF) in an experimental model of neointima hyperplasia. The results showed that the antioxidant effect is mediated by the downregulation of Lectin-like oxidized low-density lipoprotein receptor-1 (LOX-1) receptor and ROS formation that led to a regression of artery injury [105]. The restoration of the total oxidative status has also been confirmed after oral administration of the bergamot polyphenol fraction (BPF), derived from juice and albedo, in an animal model of metabolic associated fatty liver disease (MAFLD). Specifically, it was associated with a reduction in specific markers of oxidative stress, such as c-Jun N-terminal kinase (JNK) and p38 MAP kinase activity, in the liver, and with the restoration of total antioxidant status, measured in the plasma [106]. The neutralization of free radical overproduction is further reflected by the amelioration of the oxidative status of the LDL profile detected in patients with metabolic syndrome. Particularly, BPF administration determined a significant re-arrangement of lipoproteins with a reduction in oxidated LDL small-size atherogenic particles and an increase in large-size antiatherogenic HDL [107]. In addition, in BPF-treated patients, a significant reduction in serum total cholesterol (TC) and LDL-C, probably due to the inhibition of HMG-CoA reductase and triglycerides (TG), was detected. Moreover, the amelioration of these parameters was associated with a significant decrease in serum glucose, transaminases, gamma-glutamyl-transferase, and inflammatory biomarkers, such as TNF-α and C-reactive protein [108]. The anti-inflammatory and lipid-lowering effects afforded by BPF were also confirmed in patients with isolated hypercholesterolemia, mixed hyperlipidaemia (hypercholesterolemia plus hypertriglyceridemia) as well as in patients with mixed hyperlipidaemia and hyperglycaemia [108], suggesting a potential use in the management of T2DM. In accordance with this hypothesis, BPF enriched with a *Cynara cardunculus* extract has shown a beneficial potentiated effect on vascular inflammation and oxidative stress in patients with T2DM and MAFLD [109], which also translated into an improvement of endothelial function and NO-mediated reactive vasodilation [109].

Finally, the BPF administration in rats revealed a very interesting effect; indeed, it can prevent heart failure through direct antioxidant action at the cardiac level, impeding myocyte apoptotic cell death. In addition, apoptosis was counterbalanced by the reduction in attrition in cardiac-resident stem cells with the consequent formation of new myocytes [110].

## 5. Conclusions

To date, the conjugation of therapies aimed to achieve the control of glucose plasma levels, and the prevention of cardiovascular disease occurring in diabetic patients, still represents a priority considering the growing incidence of hospitalization and death. In this view, a potential approach combining the use of SGLT2i and plant extracts might be considered a promising solution. Indeed, other than their hypoglycaemic effects, the molecular mechanisms underlying the cardioprotective action of SGLT2i could be potentiated through the combination with phytocomplexes able to prevent or limit the main cardiometabolic risk factors [76], thus contributing to better management of diabetic patients.
